# Detection of response to command using voluntary control of breathing in disorders of consciousness

**DOI:** 10.3389/fnhum.2014.01020

**Published:** 2014-12-23

**Authors:** Vanessa Charland-Verville, Damien Lesenfants, Lee Sela, Quentin Noirhomme, Erik Ziegler, Camille Chatelle, Anton Plotkin, Noam Sobel, Steven Laureys

**Affiliations:** ^1^Coma Science Group, Cyclotron Research Centre and Neurology Department, University of Liège and CHU Sart Tilman HospitalLiège, Belgium; ^2^Department of Neurobiology, The Weizmann Institute of ScienceRehovot, Israel; ^3^Brain Innovation B.V., MaastrichtNetherlands; ^4^Cyclotron Research Centre, University of LiègeLiège, Belgium; ^5^Department of Physical Medicine and Rehabilitation, Spaulding Rehabilitation Hospital, Harvard Medical SchoolBoston, MA, USA

**Keywords:** disorders of consciousness, breathing, sniffing, vegetative state, unresponsive wakefulness syndrome, minimally conscious state, diagnosis, brain-computer interface

## Abstract

**Background**: Detecting signs of consciousness in patients in a vegetative state/unresponsive wakefulness syndrome (UWS/VS) or minimally conscious state (MCS) is known to be very challenging. Plotkin et al. (2010) recently showed the possibility of using a breathing-controlled communication device in patients with locked in syndrome. We here aim to test a breathing-based “sniff controller” that could be used as an alternative diagnostic tool to evaluate response to command in severely brain damaged patients with chronic disorders of consciousness (DOC).

**Methods**: Twenty-five DOC patients were included. Patients’ resting breathing-amplitude was measured during a 5 min resting condition. Next, they were instructed to end the presentation of a music sequence by sniffing vigorously. An automated detection of changes in breathing amplitude (i.e., >1.5 SD of resting) ended the music and hence provided positive feedback to the patient.

**Results**: None of the 11 UWS/VS patients showed a sniff-based response to command. One out of 14 patients with MCS was able to willfully modulate his breathing pattern to answer the command on 16/19 trials (accuracy 84%). Interestingly, this patient failed to show any other motor response to command.

**Discussion**: We here illustrate the possible interest of using breathing-dependent response to command in the detection of residual cognition in patients with DOC after severe brain injury.

## Introduction

Detecting signs of consciousness is a challenging task associated with crucial implications such as subsequent care and rehabilitation, and legal and ethical decision-making. *Unresponsive wakefulness syndrome* (UWS; Laureys et al., 2010) previously referred as vegetative state (VS) is defined by wakefulness without any sign of awareness of self, or the environment (Laureys, 2005). Patients who recover from the UWS/VS will show inconsistent but purposeful behaviors suggesting the presence of conscious awareness. The minimally conscious state (MCS; Giacino et al., 2002) is now subcategorized based on the level of complexity of observed behavioral responses. MCS- is defined by non-reflexive behaviors such as visual pursuit or orientation of noxious stimuli, while MCS+ is defined by the presence of a response to command, intelligible verbalization or gestural or verbal yes/no responses (Bruno et al., 2011).

The frequency of misdiagnosis in patients with a disorder of consciousness (DOC) can be explained by the fact that reproducible goal-directed behaviors (e.g., response to command, verbalizations, visual pursuit, etc.) can be difficult to observe at the bedside (Schnakers et al., 2009). Behaviors elicited by the patients are often ambiguous, inconsistent, and constrained by varying degrees fluctuating arousal, making it very challenging to distinguish purely reflexive or automatic from voluntary responses (Majerus et al., 2005). Also, patients with severe motor impairments may fail to communicate their level of consciousness and recovery of conscious awareness may precede motor recovery in some patients (Stender et al., 2014). Diagnostic errors can be reduced by the use of standardized validated scoring tools, such as the Coma Recovery Scale–Revised (CRS–R; Giacino et al., 2004; Schnakers et al., 2008a) although misdiagnosis can still arise even with rigorous behavioral testing (Monti et al., 2010).

Therefore, repeated assessments with complementary exams using neuroimaging techniques or brain-computer interfaces (BCI) may help improving diagnostic accuracy (Lesenfants et al., 2014; Stender et al., 2014). Because of its rich innervation pattern, the ability to voluntarily sniff may remain preserved following severe brain injury (Shimokawa et al., 2005). We here tested the application of a *sniff controller*—a previously validated technique in a population of healthy controls and patients with severe motor disabilities such as locked in syndrome and quadriplegia (Plotkin et al., 2010)—as a complementary way to assess the level of consciousness at bedside for patients with DOC. This technology has the advantages of being portable, cheap and relatively simple to use without requiring extensive training.

## Material and methods

For this clinical validation study we included patients with chronic (>1 month) UWS/VS or MCS based on CRS-R assessments. Exclusion criteria were the presence of tracheotomy, administration of sedatives, previous nose injuries and suspected hearing impairment (based on medical history and the absence of an auditory startle or localization to sound on CRS-R testing). The study was approved by the ethics committee of the Faculty of Medicine of the University and University Hospital of Liège and written informed consent was obtained from all patients’ legal representatives.

The sniff controller consists of a nasal cannula that carries changes in air pressure from the nose to a pressure transducer. This transducer records the changes in nasal pressure during sniffs depending on the position of the soft palate. It then transforms these pressure changes into an electrical signal that passes to a laptop (Plotkin et al., 2010). The program used to display the transformed signal and store acquired data was written in Lab VIEW^©^ version 8.6. The proposed task was to invite the patient to try to stop a music sequence by sniffing deeply through the nose cannula. To do this, the patient had to voluntarily modulate his breathing pattern (by deeply inhaling or exhaling) to exceed the resting breathing amplitude threshold. When the threshold was exceeded, the musical sequence stopped and the trial was considered successful. The instructions at the beginning of the testing were: “We will start by recording your breathing at rest for 5 min. During this period, you have nothing to do, just breathe at ease.” The instructions for the task were: “Each time you hear a music sequence, we will ask you to deeply sniff in or out when hearing in order to stop it.” The following command: “Try to sniff in or out in order to stop the music” was repeated at the beginning of each trial. The music sequence consisted of a 30-second guitar melody. Inter-stimulus-interval (ISI) was 1-minute. After each trial and before the ISI, auditory feedback was provided by a prerecorded applause 5-second sequence for successful trials (positive feedback) and a white noise in case of unsuccessful ones (negative feedback). A minimum of 10 trials was administered. Because of the limited attentional capacities inherent to patients with DOC, the experiment was aborted after 30 min.

The normalized breathing recording was decomposed into 1 s epochs and a Hilbert transform was used to compute the envelope of the signal. The activation threshold was set to be mean + 1.5 SD of the breathing amplitude measured during 5 min resting baseline recordings prior to the experiment and allowed to detect significant (*α* = 0.05) increase of amplitude in breathing. Classification accuracy between active commands and passive ISI was evaluated on each subject. A binomial test (Müller-Putz et al., 2008) evaluated the chance level (*α* = 0.01) for each patient depending of the number of trials. The response delay was evaluated as the time required to exceed the threshold from the beginning of the command.

The signal was also individually decomposed in 20 s epochs and multitaper spectral analysis was computed on each epoch. The maximum of the frequency curve was used to calculate the respiratorion rate and corresponding amplitude. The significance of change was assessed with a Mann-Whitney *U* test (*α* = 0.05). All analyses were corrected for multiple comparisons and performed using MATLAB (The Mathworks, Inc).

## Results

From an initial cohort of 30 patients with DOC, 5 were excluded because their level of vigilance could not be sustained for the minimum of 10 consecutive trials. The final cohort consisted of 25 patients with chronic DOC (10 women; aged 33 ± 13 years; interval since insult: 31 ± 27 months), etiologies were traumatic (*n* = 15), non-traumatic (*n* = 5) or mixed traumatic/anoxic etiologies (*n* = 5); UWS (*n* = 11), MCS (*n* = 14) (see Table 1 for more details on patients’ demographic and clinical data).

**Table 1:** **Demographic, clinical and task-related data of the patients’ sample**.

				Breathing		Performance	
Patient	Age (gender)	Time since injury in months	Etiology	Mean Rate (cycles/min)	Mean Amplitude (mV)	Accuracy* (%)	Nb of trials
UWS/VS 1	24 (M)	11	TBI	18.7	0.13	46	23
UWS/VS 2	67 (F)	46	Subarachnoid hemorrhage	25.7	0.04	50	18
UWS/VS 3	31 (M)	27	TBI	30.4	0.08	59	11
UWS/VS 4	48 (F)	16	Cardiac arrest	25.8	0.12	47	20
UWS/VS 5	26 (M)	16	TBI	9.4	0.01	35	17
UWS/VS 6	34 (M)	44	TBI	9.5	0.04	55	11
UWS/VS 7	34 (M)	34	TBI	23.4	0.09	48	13
UWS/VS 8	29 (F)	2	Subarachnoid hemorrhage	21.1	0.20	50	14
UWS/VS 9	25 (M)	42	TBI	32.8	0.28	50	13
UWS/VS 10	24 (F)	22	TBI	10.3	0.52	35	23
UWS/VS 11	22 (M)	8	TBI	25.8	0.04	44	12
**MCS- 1**	**36 (F)**	**18**	**TBI**	**14.1**	**0.10**	84*	**19**
MCS- 2	38 (F)	17	TBI	18.7	0.07	47	49
MCS- 3	5 (F)	36	TBI/anoxic	35.1	0.08	62	13
MCS+ 1	37 (F)	9	Cardiac arrest	9.4	0.03	41	11
MCS+ 2	46 (M)	18	TBI	25.8	0.01	50	14
MCS+ 3	24 (M)	90	TBI/anoxic	9.4	0.03	50	10
MCS+ 4	11 (M)	49	Cardiac arrest	9.3	0.08	50	17
MCS+ 5	30 (M)	13	TBI/anoxic	21.1	0.04	50	17
MCS+ 6	23 (M)	67	TBI/anoxic	9.4	0.02	65	13
MCS+ 7	54 (F)	6	TBI	9.4	0.06	50	12
MCS+ 8	30 (M)	106	TBI/anoxic	23.4	0.02	46	22
MCS+ 9	50 (M)	8	TBI	23.4	0.03	46	12
MCS+ 10	30 (F)	4	TBI	21.1	0.06	50	11
MCS+ 11	31 (F)	66	TBI	18.8	0.06	46	36

**Figure 1:**
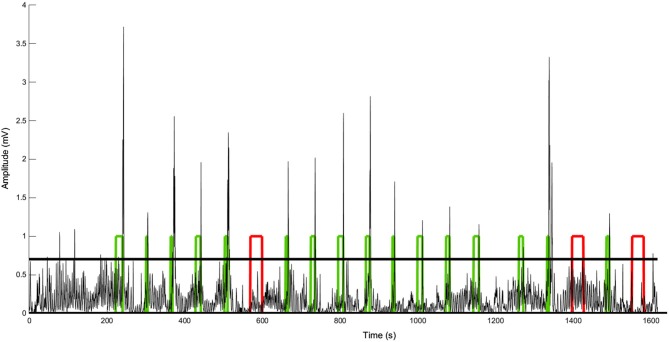
**Respiration amplitude changes over time for patient MCS-1. The horizontal bold line represents the threshold (mean + 1.5 SD of 5 min baseline recording)**. The square waves represent the periods of music presentation (acoustic guitar playing). The patient was instructed to breathe deeply (i.e., sniff) as soon as the music was presented. When this command was successfully performed (green blocks) the patient received a positive auditory feedback (i.e., the music stopped and applause was presented). Otherwise, the music continued for 30 s (red blocks) and a negative feedback was given (white noise). Note that a response to command (i.e., sniff) was observed in 16 out of the 19 trials.

We observed no differences between UWS/VS and MCS for baseline respiratory rate (16 ± 11 vs. 18 ± 8 cycles/min) and breathing amplitude (0.12 ± 0.14 vs. 0.06 ± 0.04 mV). None of the patients with UWS/VS showed a command-related change in breathing amplitude (i.e., sniff). Only one out of 14 patients with MCS showed a sniff-related response to command in 16/19 trials (accuracy 84%, chance level estimated at 71% using binomial testing with *α* = 0.01; see Figure 1 and Table 1; patient MCS-1). Response delays for this patient ranged between 4 s and 26 s (11 ± 5 s).

## Discussion

The present feasibility study illustrates that an automated user-independent breathing (i.e., sniffing)-based response to command assessment can be performed in severely injured patients with DOC. In the present sample of 30 patients with chronic DOC, 5 needed to be excluded because of fatigue (17%). None of the included UWS/VS patients showed any sign of voluntary sniffing and only 1 patient with MCS could perform the test. Interestingly, this patient only showed eye tracking (i.e., was MCS-) but showed a response to command (i.e., evolved to MCS+) on follow-up CRS-R testing. It should be noted that none of the 11 patients with MCS+ could perform the sniffing-test, while clinically showing a response to command with CRS-R testing, illustrating a 100% false negative rate.

Regarding the possibility of false positive findings, our evaluation of performance during the command and the inter-command periods allowed taking into account non-voluntary fluctuations or reflexive increases in breathing caused by an auditory startle response. The observed low sensitivity and specificity could partially be explained by the complexity of the task. Indeed, this kind of protocol requires high cognitive abilities such as sustained attention and task switching. This limitation has also been previously observed in motor-independent brain computer interface paradigms (Chatelle et al., 2012).

Recent functional neuroimaging studies based on mental imagery tasks provided evidence for awareness in patients diagnosed with UWS/VS or MCS as they presented with volitional control of brain functions detected with electroencephalography (Schnakers et al., 2008b; Cruse et al., 2011; Goldfine et al., 2011) or functional magnetic resonance imaging (Owen et al., 2006; Boly et al., 2007; Monti et al., 2010; Bardin et al., 2011). Additionally, electromyography (Bekinschtein et al., 2008) or pupil dilation (Stoll et al., 2013) monitoring can offer alternative ways to identify response to command in DOC. All of these techniques have been shown to suffer from high false negative rates and low sensitivity similar to the current results obtained with the sniff-controller (Chatelle et al., 2014). Future research should consider the use of tasks requiring less demanding attentional workload. Vigilance markers could also be considered to objectively assess and characterize performance changes over time, especially relevant in the clinical context of severely brain damaged patients (Majerus et al., 2005). It should be noted that the generalizability of our current findings in DOC (and of the Plotkin et al. study in LIS) is limited by the small number of subjects tested. Patients with prolonged DOC also often show more variable and multi-focal brainstem involvement than is the case for the well-described focal brainstem damage observed in classical LIS patients.

Typical BCI either use invasive methods (Brumberg et al., 2010) or EEG in combination with machine learning techniques (Birbaumer et al., 2008) and with few exceptions (Hill et al., 2006) training with the individual is required. Voluntary breathing modulation offers an alternative path, reflecting cognitive activity that is relatively easy and low-cost to measure for daily use, requiring nothing but a portable, easily transportable pressure transducer. This pilot study demonstrates the sniffer-controller’s potential usefulness as an additional diagnostic tool to assess a patient’s state of awareness. In selected patients, changes in breathing could also be used for single-switch communication, similarly to Müller-Putz et al. (2008); Plotkin et al. (2010); Stoll et al. (2013).

Finally, our pilot data do not show differences in respiratory rate between patients with UWS/VS and MCS. Heart rate variability analyses have previously been used to assess residual or emerging (higher level) function in brain-injured patients with DOC and even to predict outcome (Riganello et al., 2012). Future studies should evaluate long-term respiratory rate during recovery of consciousness.

In conclusion, we here report proof-of-principle for a “sniff-controller” as means of automated response to command detection in severely motor impaired patients with chronic DOC. With no training and an online user-independent system providing direct feedback to the patient, an acceptable accuracy of 84% was reached in a patient failing to show any bedside sign of command-following. However, the technique suffers from a low sensitivity and requires preserved sustained vigilance, similar to our previously proposed pupil-based method (Stoll et al., 2013).

## Authors contribution

Steven Laureys, Vanessa Charland-Verville, Lee Seal, Anton Plotkin and Noam Sobel designed the study. Vanessa Charland-Verville, Damien Lesenfants and Steven Laureys wrote the manuscript. Vanessa Charland-Verville, Damien Lesenfants, Quentin Noirhomme and Erik Ziegler conducted the analyses. Vanessa Charland-Verville, Camille Chatelle and Lee Sela collected all data. Erik Ziegler, Camille Chatelle, Quentin Noirhomme, Anton Plotkin, Noam Sobel, Lee Sela provided conceptual input and contributed to the final manuscript. All authors approved the final version of the manuscript.

## Conflict of interest statement

The authors declare that the research was conducted in the absence of any commercial or financial relationships that could be construed as a potential conflict of interest.

## Abbreviations

Unresponsive wakefulness syndrome/vegetative state: (UWS/VS)
minimally conscious state: (MCS)
minimally conscious state: MINUS (MCS-)
minimally conscious state: PLUS (MCS+)
disorders of consciousness: (DOC)
brain-computer interface: (BCI).
